# Tumor necrosis factor inhibitors for pediatric patients with SAPHO syndrome associated with acne conglobata

**DOI:** 10.1186/s12969-022-00749-9

**Published:** 2022-10-12

**Authors:** Shengyan Liu, Xia Wu, Yihan Cao, Zhaohui Li, Yuchen Liu, Mingwei Ma, Chen Li

**Affiliations:** 1 School of Clinical Medicine, Peking Union Medical College, Beijing, 100730 China; 2 Peking Union Medical College Hospital, Chinese Academy of Medical Sciences & Peking Union Medical College, Beijing, 100730 China; 3 Department of Internal Medicine, Peking Union Medical College Hospital, Chinese Academy of Medical Sciences & Peking Union Medical College, Beijing, 100730 China; 4 Department of Radiology, Peking Union Medical College Hospital, Chinese Academy of Medical Sciences & Peking Union Medical College, Beijing, 100730 China; 5 Department of Rheumatology, Fangshan Hospital Beijing University of Chinese Medicine, Beijing, 102401 China

**Keywords:** Acne conglobata, Tumor necrosis factor inhibitor, SAPHO syndrome

## Abstract

Synovitis, acne, pustulosis, hyperostosis, and osteitis (SAPHO) syndrome is a rare chronic inflammatory disease with osteoarticular and cutaneous involvements as its main manifestations. Acne conglobata as a severe acne form may affect adolescent patients. Tumor necrosis factor inhibitors are usually used as a second-line therapy for refractory SAPHO syndrome and has been reported to treat acne conglobata successfully. We herein report three cases of adolescent patients with SAPHO syndrome associated with acne conglobata who were successfully treated with TNFi therapy.

**Supplementary Information:**

The online version contains supplementary material available at 10.1186/s12969-022-00749-9.

Dear editor:

Synovitis, acne, pustulosis, hyperostosis, and osteitis (SAPHO) syndrome is a rare chronic autoinflammatory disease with osteoarticular and dermatological involvement. Palmoplantar pustulosis and severe acne are the most common types of cutaneous manifestations. As a severe form of nodular acne vulgaris, acne conglobata usually presents with deep burrowing abscesses interconnected with each other and primarily affects adolescent boys with SAPHO syndrome. If not treated timely, acne conglobata may cause disfiguring scars [1]. Tumor necrosis factor inhibitors (TNFi) have been used as a second-line therapy to treat refractory SAPHO syndrome [2]. Moreover, case reports have also recorded improvement of acne conglobata in response to TNFi [3]. Herein, we report three cases of adolescent patients with SAPHO syndrome associated with acne conglobata who were successfully treated with TNFi therapy.

These patients’ baseline data were gathered before and during a 12-week followed-up (Table 1). All the patients and their legal guardians have signed the written informed consents. Before the follow-up, These patients were treated with NSAIDs or other therapies before but didn’t show significant disease remission. Two patients were treated with TNFi before and gained good efficacy, but acne conglobata and osteoarticular pain worsened quickly after reducing the dosage.

**Table 1:** Baseline data of the 4 patients with SAPHO syndrome

Patient No.	1	2	3
Age, yrs./sex	14/M	14/M	15/M
Disease duration, months	48	0	3
Lesions revealed by whole-body bone scintigraphy	Right mandible	Bilateral sternoclavicular joints, hip joints and sacroiliac joints	Sternum, T8, T10, L4, L5, left hip joint, left femur, right ischium
Dermatological manifestation	Nodules, drainage incisions and scars on cheeks, lower jaw, upper arms and chest	Nodules, drainage incisions, sinus tracks and scars on forehead, cheeks, upper back and buttocks	Nodules, drainage incisions and scars on forehead, cheeks, back and chest
Prior treatments	NSAIDs, antibiotics, TNFi,	NSAIDs, TGP	NSAIDs, minocycline, SASP, TNFi, BPs
TNFi therapy	Etanercept (50 mg, qw)	Etanercept (50 mg, qw)	Adalimumab (40 mg, q2w)
Clinical scores			
Pain VAS	0	2	2
BASDAI	0.8	0.8	1.0
BASFI	0.3	0.5	5.1
ASDAS	1.2	3.1	3.4
HAQ-S	0	0.125	3.125
DLQI	6	2	6
Laboratory findings			
ESR, mm/h	5	26	54
CRP, g/L	5.03	30.7	32.7

*NSAIDs* Nonsteroidal anti-inflammatory drugs, *TGP* Total glycosides of paeony, *SASP* Salicylazosulfapyridine, *BPs* Bisphosphonates, *VAS* Visual analog scale, *BASDI* Bath ankylosing spondylitis disease activity index, *BASFI* Bath Ankylosing Spondylitis Functional Index, *ASDAS* Ankylosing Spondylitis Disease Activity Score, *HAQ-S* Health assessment questionnaire for the spondylarthropathties, *DLQI* Dermatology life quality index

After 12 weeks of TNFi treatment, the acne conglobata condition improved (Supplementary Figs. 1, 2 and 3). The clinical scores and laboratory indexes decreased significantly (Fig. 1), indicating the alleviation of manifestations and inflammatory condition, especially in case 3. Patient 2 and patient 3’s osteoarticular involvements were more extensive at the beginning. They were reported to have released anterior chest wall and hip joints pain, in addition to the significantly declined clinical scores. In terms of adverse events, none were observed other than increased uric acid. Although the pathogenesis of SAPHO syndrome remains unclear, there is a theory that proinflammatory cytokines such as IL-8 and TNF-α can be triggered by *Cutibacterium acnes* infection, leading to skin lesion such as acne conglobata as well as a systemic inflammatory response [4]. Commonly used TNF-α antagonists include infliximab, etanercept and adalimumab, all of which have been reported to successfully alleviate SAPHO syndrome [5]. To our knowledge there are no other case series describing the efficacy of TNF α antagonists treatment for patients with acne conglobate in SAPHO syndrome. Interestingly, Su et al. found in a 42-year-old male patient with SAPHO syndrome that after treatment with etanercept, bone and joint lesions were improved like the 3 patients in this case series, while skin lesions were not significantly alleviated, possibly because TNFi could not treat the permanent damage of hair follicular in acne aggregata [6]. Therefore, TNFi treatment for SAPHO should be considered earlier on in the disease and continued to maintain remission. For the safety profile, hyperuricemia was observed in two patients. This is not a known adverse event for TNFi and has not been reported in patients with SAPHO. The specific relation between hyperuricemia and the treatment is unclear, and the safety of TNFi treatment still needs to be verified by large-scale clinical trials. It is a descriptive observation with small sample size and short follow-up period, but the results are promising and should encourage further study to confirm the efficacy and safety profile. Overall, long-term TNFi therapy has the potential to be a favorable option for adolescent SAPHO syndrome patients with acne conglobata.

**Fig. 1:**
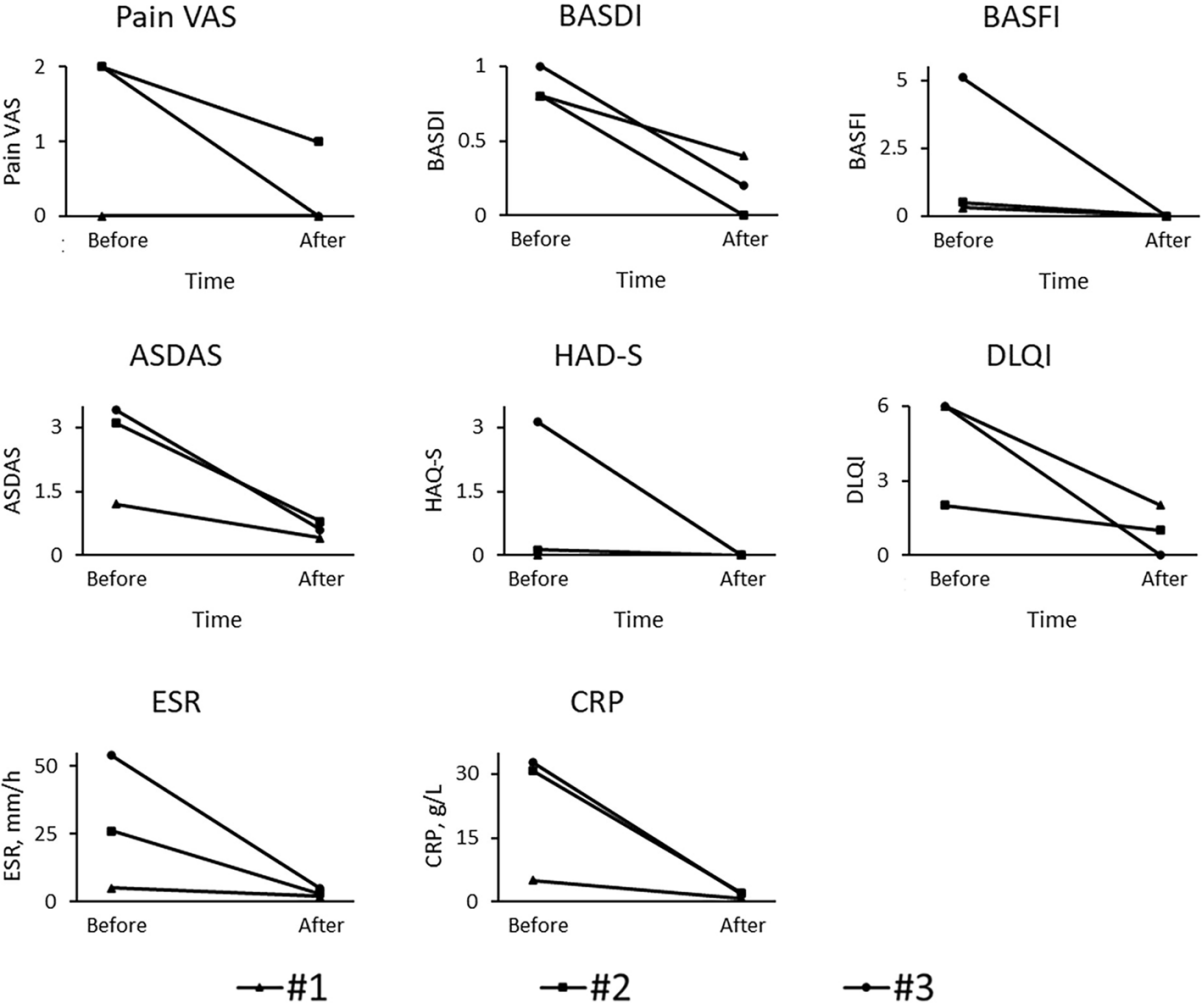
Clinical scores and laboratory indexes before and after the 12-week TNFi treatment

## Supplementary Information

**Additional file 1: Supplementary Figure 1.** Skin lesions of patient 1 before (A) and after (B) the 12-week TNFi treatment. The severe acne characterized by cyst nodules and abscesses in the cheeks improved after the treatment. The area of the acne shrank.

**Additional file 2: Supplementary Figure 2.** Skin lesions of patient 2 before (A) and after (B) the 12-week TNFi treatment. The severe acne characterized by cyst nodules and abscesses in the forehead and nose improved after the treatment, left only some slight scar formation.

**Additional file 3: Supplementary Figure 3.** Skin lesions of patient 3 before (A) and after (B) the 12-week TNFi treatment. The severe acne characterized by cyst nodules and abscesses in the forehead, nose and cheeks improved after the treatment, left only some superficial scar formation.

## Data Availability

All data generated or analysed during this study are included in this published article.

## Abbreviations

SAPHO: Synovitis, Acne, Pustulosis, Hyperostosis, and Osteitis
TNFi: Tumor necrosis factor inhibitors
NSAIDs: Nonsteroidal anti-inflammatory drugs
TGP: Total glycosides of paeony
SASP: Salicylazosulfapyridine
BPs: Bisphosphonates
VAS: Visual Analog Scale
BASDI: Bath Ankylosing Spondylitis Disease Activity Index
BASFI: Bath Ankylosing Spondylitis Functional Index
ASDAS: Ankylosing Spondylitis Disease Activity Score
HAQ-S: Health Assessment Questionnaire for the Spondylarthropathties
DLQI: Dermatology Life Quality Index
