# Supplementary figures and images for: Tumor necrosis factor inhibitors for pediatric patients with SAPHO syndrome associated with acne conglobata
Source: Pediatr Rheumatol Online J. 2022 Oct 12;20:88. doi: 10.1186/s12969-022-00749-9 (PMC9555096; doi:10.1186/s12969-022-00749-9)

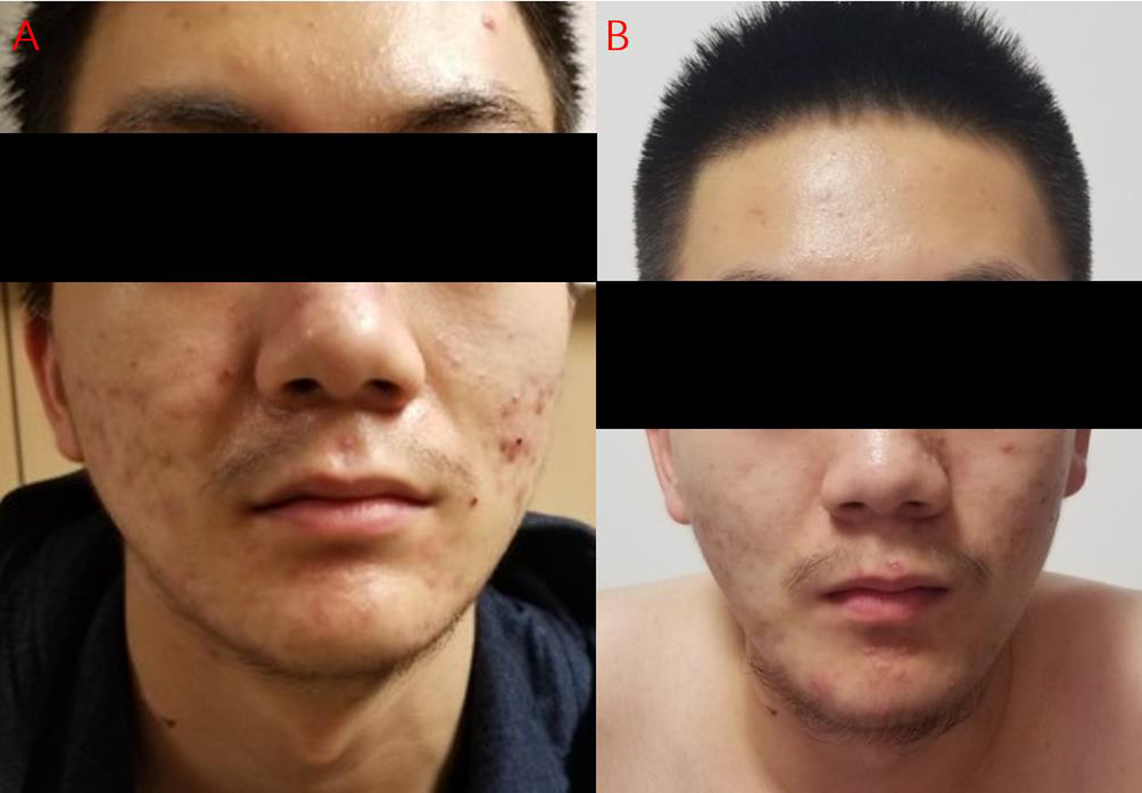

Supplement: Supplementary file 1 — Additional file 1: Supplementary Figure 1. Skin lesions of patient 1 before (A) and after (B) the 12-week TNFi treatment. The severe acne characterized by cyst nodules and abscesses in the cheeks improved after the treatment. The area of the acne shrank. [file 12969_2022_749_MOESM1_ESM.tif]

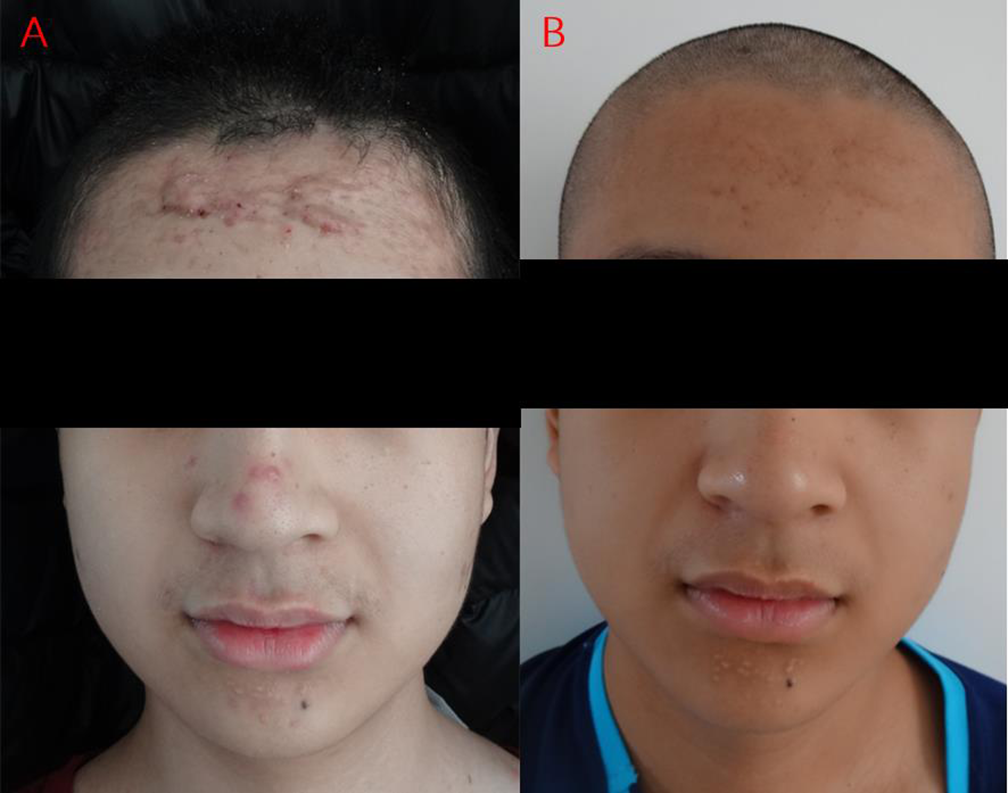

Supplement: Supplementary file 2 — Additional file 2: Supplementary Figure 2. Skin lesions of patient 2 before (A) and after (B) the 12-week TNFi treatment. The severe acne characterized by cyst nodules and abscesses in the forehead and nose improved after the treatment, left only some slight scar formation. [file 12969_2022_749_MOESM2_ESM.tif]

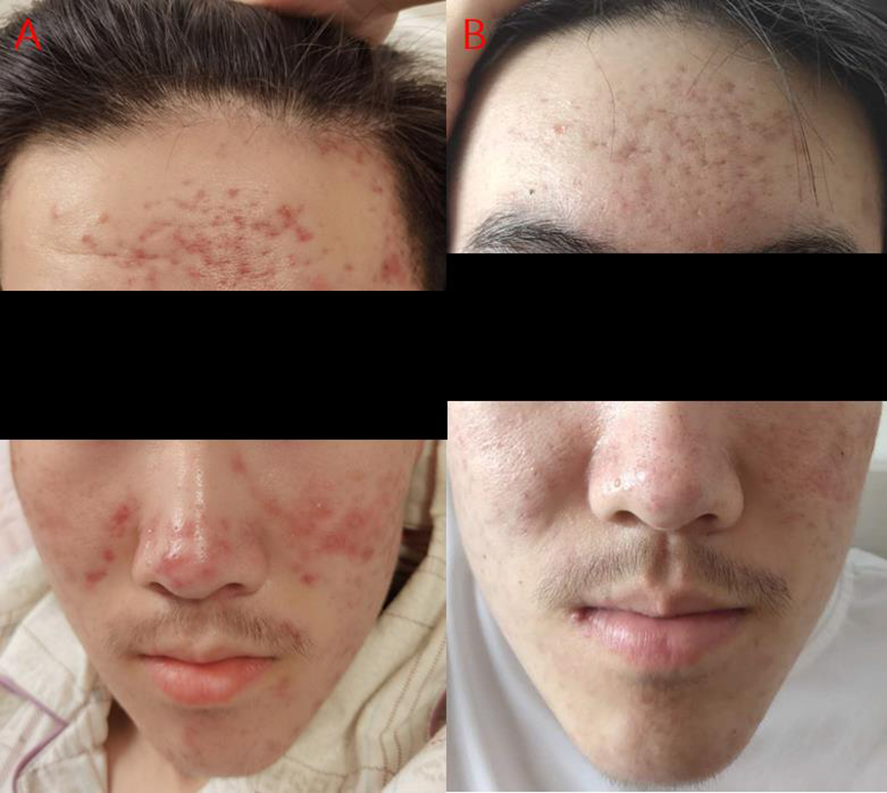

Supplement: Supplementary file 3 — Additional file 3: Supplementary Figure 3. Skin lesions of patient 3 before (A) and after (B) the 12-week TNFi treatment. The severe acne characterized by cyst nodules and abscesses in the forehead, nose and cheeks improved after the treatment, left only some superficial scar formation. [file 12969_2022_749_MOESM3_ESM.tif]
